# Comparative and stability study of glucose concentrations measured in both sodium fluoride and serum separator tubes

**DOI:** 10.1016/j.plabm.2024.e00360

**Published:** 2024-01-23

**Authors:** Mustapha Dibbasey, Solomon Umukoro, Abdoulie Bojang

**Affiliations:** Medical Research Council Unit the Gambia at London School of Hygiene and Tropical Medicine, United Kingdom

**Keywords:** Blood glucose, Sodium fluoride tubes, Serum separator tubes/serum gel tubes

## Abstract

**Introduction:**

Sodium fluoride/potassium oxalate (NaF/KOx) tubes has been regarded as the gold-standard tubes for glucose analysis. Even though their ineffectiveness in immediately inhibiting glycolysis has been reported in several studies especially in the first 1–4h, they are still used in our clinical biochemistry laboratory for glucose measurement. However, in its absence, only serum separator tubes are employed for glucose measurement. We aim to determine whether serum separator tubes (SSTs) can replace NaF/KOx tubes for laboratory measurement of blood glucose and to assess the stability of glucose concentrations for 3 days period.

**Methods and findings:**

NaF/KOx tube type was the reference method while SSTs type was the candidate method for glucose measurement. A total of 50 paired samples collected separately in NaF/KOx tubes and SSTs from healthy adult participants in the Gambia Adults Reference Intervals Study (GARIS) project were used as the project sample size. Following blood collection and separation, the glucose concentration was measured within 2 h, and at 24h, 42h and 72h time-points. Our data analysis showed no significant difference in the mean glucose concentrations between the reference tube and candidate tube types (Mean difference = 0.06 mmol/L; P = 0.38) recorded in the different timepoints. Using growth trajectory and mixed effects model, the study data further showed no significant change in the glucose concentrations (p = 0.25) for three days period.

**Conclusions:**

The study confirms that SSTs can produce similar glucose results when employed in the absence of NaF/KOx tubes. Besides, the glucose concentrations were stable in both tubes for three days when the samples were separated within 2 h and refrigerated in 2–8°C.

## Abbreviations:

SSTs: Serum Separator Tubes
NaF/KOx: Sodium fluoride/potassium oxalate
WHO: World Health Organisation
ADA: American Diabetes Association
EDTA: Ethylenediamine tetra acetic acid
GARIS: Gambia Adults Reference Interval Study
NaF: Sodium Fluoride
MRC: Medical Research Council
ATP: Adenosine Triphosphate
GARIS: Gambia Adult Reference Interval Study
HIV: Human Immunodeficiency Virus
VDRL: Venereal Disease Research Laboratory
RBCs: Red Blood Cells
≤: Less than or equal to
≥: Greater than or equal to

## 1 Introduction

Diabetes mellitus (DM) is a group of metabolic disorders characterised by chronic hyperglycaemia resulting from defects in insulin synthesis, insulin action, or both [1]. DM is a global health burden and accounts for 3.6 % of the annual health budget of The Gambia [2]. In 2014, 422 million people in the world had diabetes – a prevalence of 8.5% among the adult population and the global prevalence of diabetes is projected to increase from 171 million in 2000 to 366 million in 2030, making it the 7th leading cause of death [[3], [4]].

Laboratory measurement of blood glucose concentration is the gold-standard approach for diagnosis and management of DM as well as in the identification of patients at risk of developing DM. The increase prevalence of DM has made glucose one of the most requested biochemical analytes worldwide. Glucose is an unstable molecule in the whole blood due to the impact of glycolysis [5], a metabolic pathway which converts glucose molecule into pyruvate [6]. For many decades, NaF/KOx tubes were the gold standard collection tubes of choice when accurate glucose measurement was required. The tube type containing a combination of sodium fluoride (NaF) and potassium oxalate (KOx) was primarily designed to immediately inhibit glycolysis. However, several recent findings have criticized the effectiveness of NaF/KOx tubes in inhibiting glycolysis, especially in the first 1–4 h [[7], [8], [9]]. Mikesh and Bruns [10] indicated that complete inhibition of glycolysis by fluoride can take as long as 4 h during which time glucose can decrease as much as 0.6 mmol/L at room temperature. Gambino [11] criticized the false notion that NaF/KOx tube is an effective glycolysis inhibitor in the absence of early centrifugation following blood collection. This criticism was proven by Gambino et al. [11] study which reported that the mean glucose concentration reduced by 4.6% at 2 h and by 7.0% at 24 h when blood was drawn into NaF/KOx tubes, indicating a pre-analytical loss. Furthermore, other studies including Waring et al. [12], Turchiano et al. [13], and Shi et al. [14] have shown that early centrifugation of blood samples in lithium-heparin and SSTs produced higher mean glucose concentration than the mean glucose concentration of NaF/KOx tubes. Elleri et al. [15] reported a decrease in blood glucose by 0.47 mmol/L despite samples were collected in NaF/KOx tube and iced immediately. Based on the findings from the studies, both ADA in 2011 and WHO discourage the use of NaF/KOx tubes to control glycolysis [16].

The practical drawback of NaF/KOx tubes is not only its ineffectiveness in immediate glycolysis blockage but specificity to only measurement of glucose concentrations in blood and cerebrospinal fluid samples. However, SSTs have shown to be suitable for laboratory analysis of a wide spectrum of clinical chemistry analytes including blood glucose. The SSTs contain a gel which separates serum from cellular components, thus abrogating glycolysis. When compared to NaF/KOx tubes, a study conducted by Turchiano et al. [17] demonstrated that mean glucose concentration for SSTs where significantly higher than the mean glucose concentration for NaF/KOx tubes whilst Frank et al. [18] reported SSTs mean glucose concentration to be 1.15% lower than NaF/KOx mean glucose concentration when analysis was performed within 10 min after blood collection. Gambino [19] study demonstrated that glucose concentration in 61% of the serum samples collected in SSTs was greater than the glucose concentration in the paired plasma samples collected in NaF/KOx tubes. Following the editorial support of the study conducted by Fernandez et al. [13], which reported no difference in glucose concentrations in samples collected in both NaF/KOx tubes and SSTs in field condition and separated within 2h of blood collection, Bruns [20] questions the need for NaF/KOx tubes for blood glucose analysis. Further several studies have suggested both NaF/KOx tubes and SSTs to be suitable candidates for glucose measurement [21,[22], [23]]. Currently, SSTs type is used for laboratory measurement of blood glucose in the absence of NaF/KOx tubes in our clinical biochemistry laboratory, but its suitability is not well proven in our setting to discourage widespread use. Also in most of the studies, the samples were collected in field conditions which means there findings are not directly translatable in our clinical laboratory settings. To add, the early centrifugation approach shown to have superior outcome was not practically possible in our busy clinical settings. As such, we aim to determine whether SSTs can replace NaF/KOx tubes for laboratory-based measurement of blood glucose and to assess the stability of glucose concentrations for 3 days period in both tube types.

In this study, we have provided an insight into whether glucose concentrations are varied significantly in the paired NaF/KOx, and SSTs samples collected in the laboratory environment.

## 2 Materials and methods

### 2.1 Sample collection/handling

The study was conducted in clinical laboratory department hosted at the Kuyateh Building at the Medical Research Council Unit the Gambia at London School of Hygiene and Tropical Medicine (MRCG). The clinical laboratory of MRCG is in the Kanifing municipality, greater Banjul area, the Gambia. The Gambia is geographically situated in West Africa with a population of about 2.5 million and an area of 10,000km2. Clinical laboratory is an ISO15189:2012 accredited by KENAS accreditation body ISO15189:2012 [24].

With the permission of the study investigators, the blood samples for the practical project were obtained from the GARIS project which started in March 2015, an on-going project targeting to establish haematological and biochemical reference values for Gambian adults in Greater Banjul Area. During the practical project (1 March to April 11, 2015), a total of 50 pairs samples collected separately in NaF/KOx and SSTs from healthy adult participants in the GARIS project were used as the project sample size.

The participants who were healthy adult donors between the age of 18–45 years (≥18 and ≤ 45 years) residing in the urban areas and negative for Human Immunodeficiency Virus, Syphilis and Hepatitis B virus were enrolled into the GARIS project. The participants were bled, and the blood samples were received, in the clinical laboratories under room temperature. The first 50 pairs of GARIS samples with sufficient volume were used in the practical project.

### 2.2 Sample analysis

The glucose analytes were measured by Ortho-Clinical Diagnostics™ VITROS™ 350 System (Ortho-Clinical Diagnostics, US). Prior to the analysis, quality controls were run to ascertain the functionality of the Vitros 350 Analyser, which performed the glucose analysis of the project samples. Centrifugation (separation of plasma from NaF blood and serum from clotted blood samples) and first analysis of glucose concentrations were performed within 2h of blood collection, and then the serum and plasma samples were stored at 2–8°C temperature. The subsequent analyses were exactly performed at the following time-points: 24, 48 and 72h. The study samples were analysed using the same lot of reagents, eliminating any lot-to-lot variability in the results.

### 2.3 Statistical analysis

The statistical analysis was performed using STATA version 12 and Microsoft Word excel. Paired *t*-test was performed to determine any significant statistical difference in the mean glucose concentration recorded in the time-points. Pearson correlation was performed to determine the association between NaF/KOx tubes and SSTs glucose concentrations. A mixed effects model and growth trajectory were performed to assess the stability of glucose concentrations.

## 3 Ethical Issues

The GARIS project was approved by MRC Scientific Coordinating Committee and the Gambia Government/MRC Joint Ethics Committee prior to commencement of the study [25]. Written informed consent was obtained from study participants enrolled in the GARIS study. GARIS participants were assigned unique identification numbers for maintaining anonymity and confidentiality.

## 4 Result

**Fig. 1:**
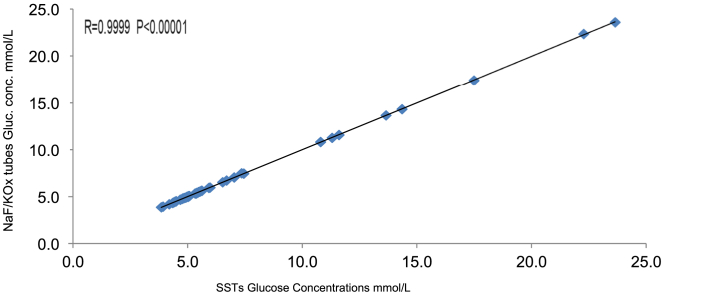
Graph showing relationship between glucose concentrations measured in NaF/KOx tubes and SSTs. SSTs: serum separator tubes. NaF/KOx: Sodium fluoride tube/potassium oxalate.

Table 1 shows mean glucose concentrations (MGC) measured in both NaF/KOx tubes and SSTs at different time-points (2h, 24h, 48h, and 72h). The overall MGC of NaF/KOx tubes (6.88 ± 4.36 mmol/L) and SSTs (6.94 ± 4.31 mmol/L) produced an overall difference of 0.06 mmol/L [Total Mean difference (NaF/KOx-SST) = 0.06 ± 0.07; n = 50]. When the MGC from the time-points were compared using two-tailed paired *t*-test, the result shows no significant difference (P = 0.38). The Pearson correlation in Fig. 1 shows high significant correlation (R = 0.9999 P < 0.00001) between NaF/KOx tubes and SSTs glucose concentrations. The P value less than 0.05 indicates a statistically significant difference.

**Table 1:** Showing MGC of NaF/KOx tubes and SSTs from different time-points.

Time-points	NaF/KOx MGC	SST MGC	MD (NaF/KOx-SST)
within 2hrs	6.86 ± 4.31	6.92 ± 4.29	−0.06
24hrs	6.88 ± 4.34	7.01 ± 4.35	−0.13
48hrs	6.87 ± 4.38	7.01 ± 4.42	−0.14
72hrs	6.92 ± 4.38	6.82 ± 4.30	0.10
**Total mean**	**6.88** ± 0.07	**6.94** ± 0.07	**−0.06**

MGC: mean glucose concentration, MD: mean difference (i.e., differences between the time-points means), SST: serum separator tubes, NaF/KOx: sodium fluoride tubes.

Furthermore, raw glucose concentrations were transformed using the inverse of the square of the glucose concentration. Post-data transformation, a mixed effects model shown in table was employed for stability study of glucose concentrations in both tubes using within 2 h as the reference category (Table 2). There was no evidence of change in the glucose concentration (overall p = 0.25) in both tubes for 3 days.

**Table 2:** Stability study of glucose concentrations from the 2h–72h of analysis.

Time-points	Estimated difference (95 % CI^a^)	p. Value
Within 2 h”	0	
24 h	0.001	0.06
48 h	0.0003	0.33
72 h	0.0002	0.67
**Time**		**0.25**^b^

a 95 % confidence interval.

b Overall P. value.

**Fig. 2:**
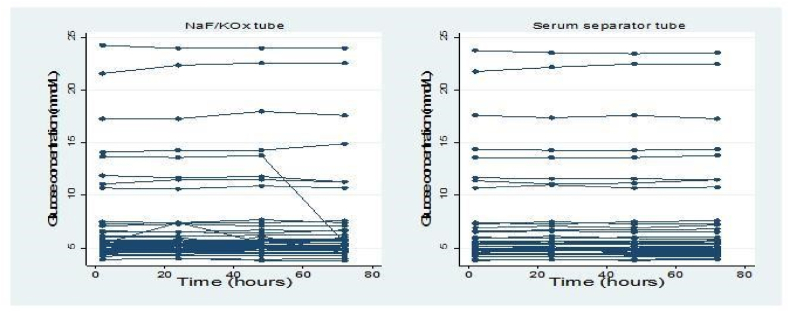
Growth trajectory graph for determining the stability of individual glucose concentrations from 2 to 72 h.

Growth trajectory graph (Fig. 2) was used to determine the stability of individual glucose concentrations in both tubes after the first analysis and up to 72h of analysis. The graph shows that the glucose concentrations measured with NaF/KOx, and SSTs were stable from the first analysis up to 72h at 2–8°C.

## 5 Discussion

The study demonstrates no significant difference in the mean glucose concentrations between NaF/KOx tubes and SSTs. The study outcome is consistent with the outcome of the studies of Fernandez et al. [13], Li et al. [21] and Al-Kharusi et al. [22], which qualify both tubes to be suitable candidates for glucose analysis. The slight increase in the SSTs MGC (0.06 mmol/L) as compared to NaF/KOx tubes explains the ineffectiveness of NaF/KOx tubes and that the use of NaF/KOx tubes may impact DM diagnosis especially for patients in borderline area of impaired glucose tolerance. Furthermore, this study, and other comparative and review studies have proven that the reference tube is ineffective in its role to instantaneously block glycolysis [5,10,11,19,20], hence its usage is being discouraged by ADA [16].

Since both tubes are suitable candidates, preference should be given to SSTs as it can be used to analyse wide spectrum of biochemical analytes and avoid inconveniencies and reduce mistakes associated with analysing both plasma and serum at the clinical biochemistry laboratory for an individual patient. Clinically, the use of only SSTs will reduce the volume of blood collected from patients when glucose and other analytes are requested together and improve glucose turn-around-time as well as laboratory workflow. Even with the low cost, elimination of NaF/KOx tubes would be a significant cost-saving measure for the National Health Service as large number of laboratory-based glucose tests are performed annually.

However, this study result differs from the previous studies including Gambino [19], Waring et al. [12], Turchiano et al. [17] and Shi et al. [14], which reported higher significant differences in the MGC of SSTs as compared to the MGC of NaF/KOx tubes. The significant difference was due to early separation as SSTs blood samples were centrifuged immediately after collection whereas NaF/KOx samples were centrifuged immediately before glucose analysis. Since blood cells (erythrocytes, leucocytes, and thrombocytes) are known to metabolise blood glucose, Mikesh and Bruns [10] indicated that early separation of plasma/serum from the blood cells instantaneously abrogates glycolysis as the blood cells are prevented from metabolising blood glucose to glucose-6-phosphate and other phosphates. Frank et al. [18] and Bhargava et al. [23] found SSTs MGC to be lower than NaF/KOx MGC when centrifugation and analysis were performed within 1h–2h after collection.

After the first analysis, glucose concentrations were stable at 2–8°C in both tubes for three days. Similar findings were reported by Chan, Swaminathan and Cockram [7], Cuhadar et al. [26] and Al-Kharusi et al. [22] even though the samples were stored in room temperature [[7], [26]^22^]. The stability of glucose concentrations after 4h in NaF/KOx tubes was explained in the study of Mikesh and Bruns [10] which reported that NaF inhibits enolase within 5min of addition to the blood while enzymes in the upstream of glycolysis pathway remain active, allowing continuous metabolism of glucose by blood cells into glucose-6-phosphate and other phosphorylated metabolites until there is no available supply of ATP in the cells. Supply of ATP is exhausted about 60 min after addition of NaF and then glucose concentration becomes stabilised in the tube [5]. In the case of SSTs, Bruns [20] explains that SSTs contains gel barrier that separates blood cells from plasma, thus this means that glycolysis is completely ceased as soon as separation is performed. It further explains the stability of glucose concentrations in the SSTs [10,14]. The stability study hence proves that glucose concentration can be measured in plasma/serum within 3 days when the samples are refrigeration at 2–8 °C.

The study failed to address potential confounders such as diabetes status that can influence the observed results. From the study of Spencer et al. [27], the data showed that plasma glucose in NaF/KOx reduced by 16.4 in non-diabetic and by 10.4 in diabetic patients.

Furthermore, it was not possible to estimate the effects of red blood cells (RBCs) haemolysis on glucose concentrations compared between the two tubes in the study. The studies of Fernandez et al. [13], Al-Kharusi et al. [22] and Ko et al. [28] all reported high RBCs haemolysis in NaF/KOx tubes than in SSTs samples but failed to assess the impact of the haemolysis. This high haemolysis rate observed can potentially influence glucose concentration and might have resulted in the slight decrease in NaF/KOx mean glucose concentration by 0.06 mmol/L. Catalase released from the lysed erythrocytes can negatively affect glucose values [29]. However, we believe the potential limitations do not have significant bearing on our findings.

The study confirms that SSTs can be satisfactorily employed in the place of NaF/KOx tubes for laboratory glucose measurement in our setting. Accepting the use of SSTs for glucose measurement offers numerous operational benefits including cost-saving measure for the National Health Service. The glucose concentrations were stable in both tubes for three days post centrifugation when the plasma/serum samples were refrigerated at 2–8°C. Further study is required to provide an insight into the effects of haemolysis on the glucose concentrations and its stability whilst considering potential confounders.

## 6 Ethics approval and consent to participate

This study was approved by both Scientific Coordinating Committee and Ethics Committee of Medical Research Council the Gambia at London School Hygiene Tropical Medicine. All methods were performed in accordance with the Declaration of Helsink.

## Funding

This work was supported by the 10.13039/501100000265Medical Research Council Unit the Gambia at the 10.13039/100009660London School of Hygiene and Tropical Medicine.

## CRediT authorship contribution statement

**Mustapha Dibbasey:** Conceptualization, Data curation, Formal analysis, Methodology, Writing – original draft, Writing – review & editing. **Solomon Umukoro:** Supervision, Writing – review & editing. **Abdoulie Bojang:** Conceptualization, Funding acquisition, Supervision, Writing – review & editing.

## Declaration of competing interest

The authors declare the following financial interests/personal relationships which may be considered as potential competing interests: This study was supported by the Medical Research Council Unit The Gambia at the London School of Hygiene and Tropical Medicine.

## Data Availability

Data will be made available on request.
