# Supplementary material for: Comparative and stability study of glucose concentrations measured in both sodium fluoride and serum separator tubes
Source: Pract Lab Med. 2024 Jan 23;39:e00360. doi: 10.1016/j.plabm.2024.e00360 (PMC10832486; doi:10.1016/j.plabm.2024.e00360)
Supplement: Multimedia component 1 [file mmc1.docx]

Table S1: Summary of the results of previous findings

| **Authors** | **Year of Publication** | **Previous Findings Summary** |
| --- | --- | --- |
| Chan, A. Y. W. Swaminathan, R. and Cockram, C. S | 1989 | Glucose concentration decreased markedly (P = 0.001) beginning from the first hours of storage in plain serum while remain stable when stored at 4 ºC in gel tubes, SSTs. The finding in this study showed glucose molecule were stable when stored in chilled temperature in Serum gel (SSTs) and discouraged the use of plain serum tube (red-top). |
| Roux, C. W. L. Wilkinson, S. D. Pavitt, D. V. Muller, B. R. and Alaghband-Zadeh, J. | 2004 | Plasma glucose concentrations obtained from blood collected into tubes containing glyceraldehyde, sodium fluoride (NAF) and potassium oxalate tubes will more closely reflect those of the patient at venepuncture. Tube containing glyceraldehyde plus sodium fluoride and potassium oxalate are superior to existing NAF tube that contains only sodium fluoride. |
| Lippi, G. Salvagno, G. L. Montagnana, M. Brocco, G. and Guidi, G. C. | 2006 | The study showed that pre-analytical haemolysis affect final glucose concentration and the study also showed the haemolysis to be more common NAF than SSTs. |
| Waring, W. S. Evans, L. E. and Kirkpatrick, C. T. | 2007 | The finding showed mean glucose concentration in SST serum samples was 5.2 mmol/l whereas the concentration in tubes containing NaF–KOx was 4.9 mmol/l, further emphasis the suitability of SSTs for glucose analysis |
| Mikesh, L. M. and Bruns, D. E. | 2008 | The study showed NAF tube do not abrogate glycolysis immediately after blood collection but keeping the samples in ice abrogated glycolysis immediately after blood collection. |
| Elleri, D. Acerini, C. L. Allen, J. M. Larsen, A. F. Wilinska, M. E. Dunger, D. B. and Hovorka, R. | 2009 | The study investigated the effect of a 3-h time-lag between blood sampling and glucose measurement in type 1 diabetes in NAF Tube. The study showed blood glucose decreased by 0.47 mmol/L despite samples being collected in NAF tubes and placed on ice. |
| Gambino, R. Piscitelli, J. Ackattupathil, T. A. Theriault, J. L. Andrin, R. D. Sanfilippo, M. L. and Etienne, M. | 2009 | The mean glucose concentration decreased by 0.3% at 2h and by 1.2% at 24h when blood was drawn into tubes containing citrate buffer, NaF, and EDTA. In contrast, the mean glucose concentration decreased by 4.6% at 2 h and by 7.0% at 24 h when blood was drawn into tubes containing NaF and sodium oxalate. Hence, the study recommended addition of citrate buffer to NAF tube for obtaining an accurate glucose concentration as NAF is not abrogating glycolysis. |
| Shi, R. Z. Seeley, E. S. Bowen, R. and Faix, D. J. | 2009 | The mean glucose concentrations for NaF-KOx samples and Li-Heparin samples were 5.7 mmol/l and 6.1 mmol/l, respectively, with a mean difference of 0.39 mmol/l. The overall finding showed rapid separation of heparinized blood is superior to NAF tube alone for abrogating glycolytic effects on blood glucose measurements in the clinical laboratory |
| Spencer, N. C.O. Sunday. J. J. Erifeta, O. K. Georgina, O. Agbor, A. A. Esosa, U. S. and | 2011 | The finding showed that Fluoride oxalate had a better stabilizing effect on plasma glucose within the first 30 minutes while Heparin is a better stabilizer at after 120 min. The study also showed that fasting blood glucose in Lithium heparin, EDTA and Fluoride oxalate decreased at mean percentage values of 5.9%, 6.0% and 5.3% for diabetic blood samples while decreased at mean percentage values of 24.6%, 10.9% and 5.0% respectively for non-diabetic blood samples |
| Fernandez, L. Jee, P. Klein, M. Fischer, P. Perkins, S. L. Brooks, S. P. J. | 2012 | The study findings showed that in the absence of haemolysis, no effect of tube types used (NAF and SSTs) on serum/plasma glucose concentrations. The study showed that both NAF tubes and SSTs can be used under survey collection and processing conditions to measure glucose with no significant difference in reported results. |
| Frank, E. A. Shubha, M. C. and D’Souza, C. J. M. | 2012 | The study reported that mean SST serum glucose gave values lower than NAF plasma by 1.15%. SSTs glucose concentrations were less stable than NAF glucose concentration in a room temperature |
| Turchiano, M. Nguyen, C. Fierman, A. Lifshitz, M. and Convit, A. | 2012 | The study findings showed the use of NAF tube as a stabilizer of blood glucose levels results in a statistically significant 4.2% reduction in blood glucose values when compared to those collected in SSTs and centrifuged within 20 minutes of collection. The study also showed early centrifugation superiority over the use of NAF tube. |
| Li, G. Cabanero, M. Wang, Z. Wang, H. Huang, T. Alexis, H. Eid, I. Muth, G. and Pincus, M. R. | 2013 | The glucose levels in the SST and NAF tubes were statistically indistinguishable from one another but not in the SST and HEPARIN tubes and in the NAF and heparin tubes. These results suggest that red-top tubes with SST or NAF tubes with a fluoride glycolysis inhibitor may be used for reproducible glucose determinations but not Heparin. |
| Peake, M. J. Bruns, D. E. Sacks, D. B. Horvath, A. R. | 2013 | The only study the prove continuing metabolism of glucose in NAF tube despite inhibition of the downstream target enzymes inhibited by fluoride. Disapproving the notion that NAF can stop glycolysis within the first 2hours after collection |
| Al-Kharusi, A. Al-Lawati, N. Al-Kindi, M. and Mula-Abed, W. | 2014 | There is no significant difference in glucose values collected from plasma NaF tubes and serum SST, and so SST can be used in hospital laboratory settings |
| Ko, D. Won, D. Jeong, T. Lee, W. Chun, S. and Min, W. | 2015 | This study showed that the incidence of hemolysis was slightly higher for plasma sample tube (PST) than SST, although both were <1%, indicating that PST is more prone to pre-analytical haemolysis. |
| Bhargava, M., Singh, N.P. & Gupta, A.K. | 2019 | In the present study, we found that once the serum is separated from the red cells, the glucose level remained stable for at least 4 h in both tube types. The study also showed some statistical difference in NAF and SST glucose values if centrifugation is delayed, the more the delay in centrifugation the more the SSTs glucose values will be negatively affected. |
